# Ancient human mitochondrial DNA and radiocarbon analysis of archived quids from the Mule Spring Rockshelter, Nevada, USA

**DOI:** 10.1371/journal.pone.0194223

**Published:** 2018-03-09

**Authors:** Scott D. Hamilton-Brehm, Lidia T. Hristova, Susan R. Edwards, Jeffrey R. Wedding, Meradeth Snow, Brittany R. Kruger, Duane P. Moser

**Affiliations:** 1 Division of Earth and Ecosystem Sciences, Desert Research Institute, Las Vegas, NV, United States of America; 2 Department of Microbiology, Southern Illinois University Carbondale, Carbondale, IL, United States of America; 3 Department of Anthropology, University of Nevada, Las Vegas, NV, United States of America; 4 Department of Anthropology, University of Montana, Missoula, MT, United States of America; 5 Division of Hydrologic Sciences, Desert Research Institute, Las Vegas, NV, United States of America; Universitat Pompeu Fabra, SPAIN

## Abstract

Chewed and expectorated quids, indigestible stringy fibers from the roasted inner pulp of agave or yucca root, have proven resilient over long periods of time in dry cave environments and correspondingly, although little studied, are common in archaeological archives. In the late 1960s, thousands of quids were recovered from Mule Spring Rockshelter (Nevada, USA) deposits and stored without consideration to DNA preservation in a museum collection, remaining unstudied for over fifty years. To assess the utility of these materials as repositories for genetic information about past inhabitants of the region and their movements, twenty-one quids were selected from arbitrary excavation depths for detailed analysis. Human mitochondrial DNA sequences from the quids were amplified by PCR and screened for diagnostic single nucleotide polymorphisms. Most detected single nucleotide polymorphisms were consistent with recognized Native American haplogroup subclades B2a5, B2i1, C1, C1c, C1c2, and D1; with the majority of the sample set consistent with subclades C1, C1c, and C1c2. In parallel with the DNA analysis, each quid was radiocarbon dated, revealing a time-resolved pattern of occupancy from 347 to 977 calibrated years before present. In particular, this dataset reveals strong evidence for the presence of haplogroup C1/C1c at the Southwestern edge of the US Great Basin from ~670 to 980 cal YBP, which may temporally correspond with the beginnings of the so-called Numic Spread into the region. The research described here demonstrates an approach which combines targeted DNA analysis with radiocarbon age dating; thus enabling the genetic analysis of archaeological materials of uncertain stratigraphic context. Here we present a survey of the maternal genetic profiles from people who used the Mule Spring Rockshelter and the historic timing of their utilization of a key natural resource.

## Introduction

**Fig 1:**
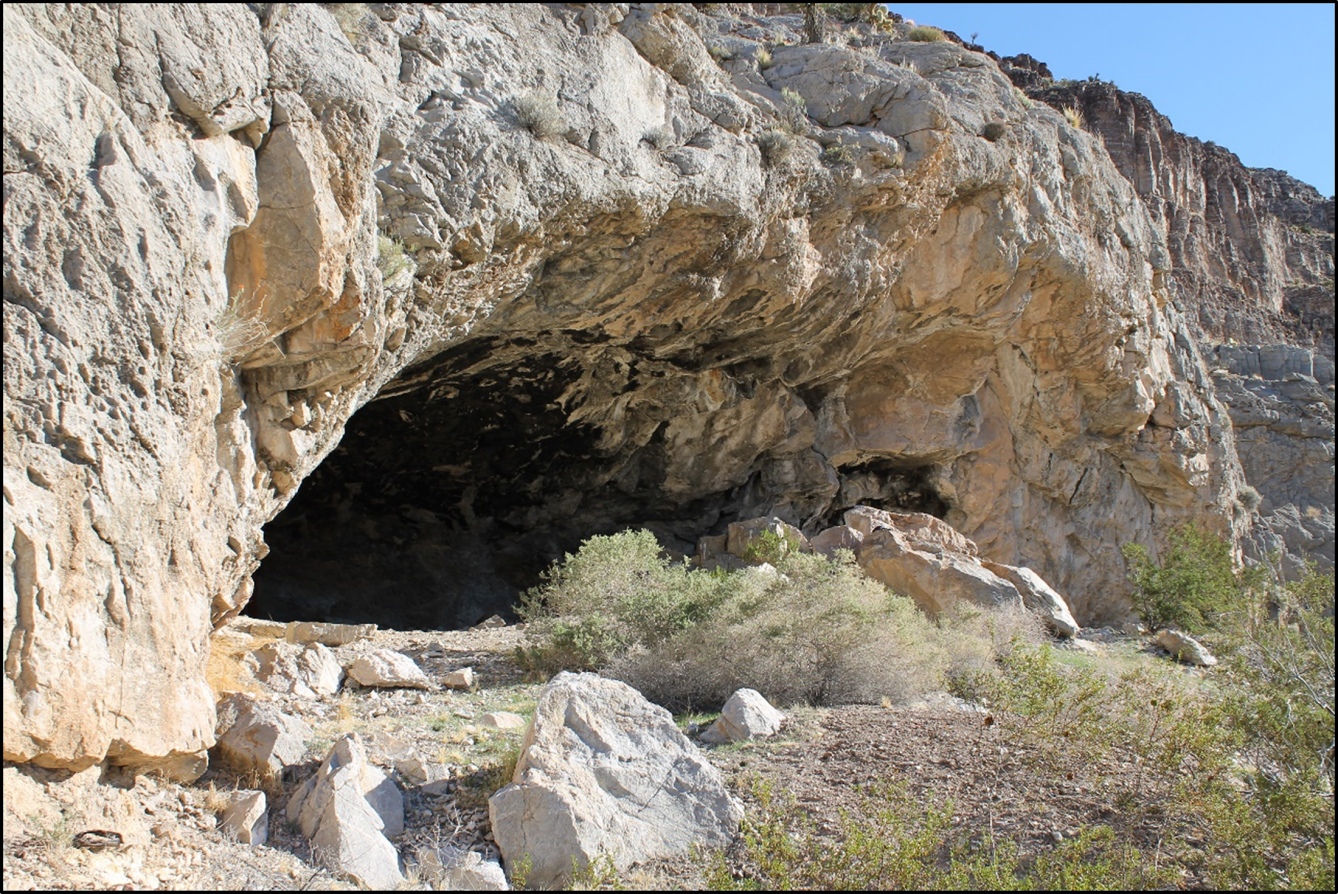
Mule Spring Rockshelter site. Photograph of the Mule Spring Rockshelter opening and apron as it appeared in 2016.

Mule Spring Rockshelter (MSR, **Fig 1**) is located at the southern boundary of North America’s hydrographic Great Basin, in the southwestern foothills of the Spring Mountain Range, near Pahrump, Nevada, USA [1]. Situated in a dolomite outcrop at 1,463 m above mean sea level, the MSR site falls within the Blackbrush (*Coleogyne ramosissima*) Plant Community of the Desert Resource Ecozone [2], which is in part characterized by varieties of agave and yucca. Consistent dry conditions, as found at MSR, can augment the preservation of perishable artifacts, and in some cases ancient DNA (aDNA [3–9]). In recent years, this has enabled DNA-based analyses, commonly applied to ancient biological remains from archaeological sites, to expand understanding of the diversity, distribution, and health of ancient peoples.

During the Late Holocene Epoch, a common food gathering practice of Great Basin inhabitants involved the processing of agave or yucca root by roasting and chewing of the plant’s inner pulp. As the stringy fibers of this material are indigestible by humans, they were expectorated as discrete wads commonly known as “quids.” Consequently, quids are sometimes found in caves and rockshelters of the region. LeBlanc *et al*. recognized quids for their potential to serve as a source of human genetic information, which may be used to further inform about the movement of ancient populations [3]. Hence, quids can be viewed as a prehistoric analog of the cheek swab used in modern genetic and ancestry testing. This is possible because of their intimate contact with the user’s mouth, allowing human cells to become entrained within aggregates of fibers of the expectorated plant material [3]. The current study was conceived to test if aDNA was recoverable from MSR quids, and whether this DNA is of suitable quality for Native American genetic analysis. The mitochondrial genome (‘mitogenome’, mtDNA) is of particular utility in aDNA studies because it provides a specific, non-recombining maternal lineage [10–21]. The analysis of single base-pair insertions, deletions, and substitutions that accumulate within the coding and control regions (which contains the hypervariable regions I and II or HVRI and HVRII) of the mitogenome; single-nucleotide polymorphisms (SNPs [22–25]), have enabled the recognition of mitochondrial haplogroups that reveal maternal ancestor-descendant relationship distributions [26–30]. In western North America, mtDNA analysis has been applied to ancient human-associated samples and was used to establish four currently recognized pan-American founder haplogroup lineages termed A2, B2, C1, and D1; correspondingly recording patterns of human migration into and throughout North and South America [7, 27, 31, 32]. Evaluation of the geographic distribution of these haplogroups across the Americas has been heavily directed by extant lineages and only to a limited degree by ancient lineages due to a paucity of preserved ancient materials [3, 7, 8, 27, 33–36]. Studies utilizing available materials have revealed a number of patterns. For example, although haplogroup C1 is broadly distributed throughout North and South America [27, 31], this haplogroup appears to have had a limited presence in the Great Basin during the late Holocene Epoch [3, 34, 37]. Surprisingly, the haplogroup diversity of the Americas from 6,000–200 years before present (YBP) does not seem to have changed overall through comparison of ancient to modern samplings, although in a few locations in the North American Southwest, diversity appears to have increased over time [15].

Contemporary Shoshone, Paiute, and Ute people maintain oral traditions of origins *in situ* [38]. Some scientific evidence suggests that Numic-speaking ancestors of contemporary native peoples moved into the Great Basin from Southern California around 950 YBP; while other evidence for the Numic presence dates back even futher [39–42]. This study contributes to our evolving understanding of the patterns and timing of movements of ancient peoples in the southwestern US and Great Basin through the time-resolved identification of populations associated with a regionally prominent rockshelter over a ~630 year period (~350 to 980 YBP). Through the detailed analysis of mtDNA sequences obtained from quid samples archived for over 50 years, the ability to provide greater detail of the Great Basin’s early genetic diversity allows for additional understanding of the evolving population in the region.

## Materials and methods

### Sample collection

MSR was excavated intermittently by the University of Nevada Las Vegas between 1967 and 1971 [1]. Among the abundant artifacts recovered at the time were over 4,000 quids. Spatial control during excavation employed the English measurement system, utilizing a 4’ x 4’ (1.2 x 1.2 meters) horizontal grid of excavation units. Shelter deposits were removed in 4” (10 cm) vertical levels and were inventoried by excavation unit and level. The quids were curated in clean brown paper bags, where they remained in atmospherically controlled museum storage until the commencement of this study.

### Quid processing and DNA extraction

**Fig 2:**
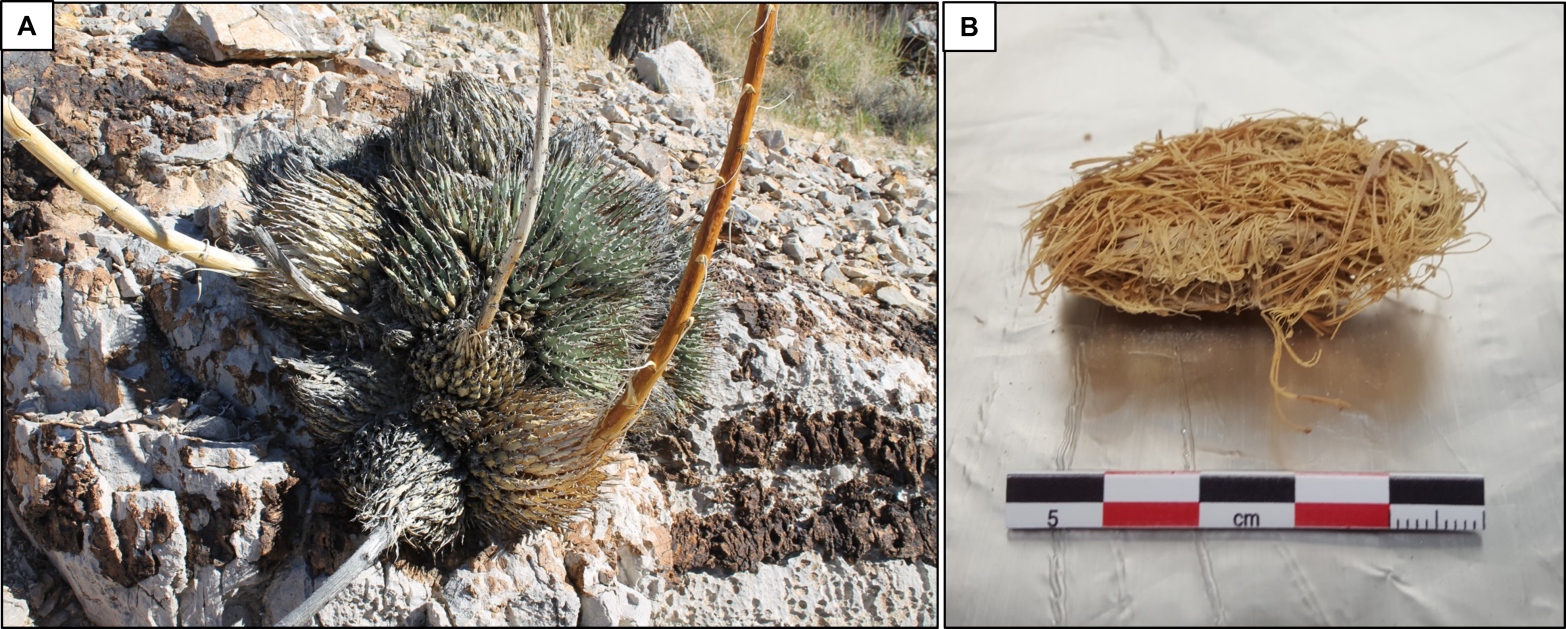
Extant agave and prehistoric quid. Example of A) an agave plant (*Agave utahensis* var. *nevadensis*) photographed near Mule Spring Cave in 2016, and B) a quid before DNA extraction (this work).

For this proof of concept study, a representative subset of 21 quids was selected from two of the 150 original excavation units (G8 and G9, **S2 Table**, [1]). Four quids each were randomly selected for detailed analysis from five of the six discrete excavation levels (0–4”, 8–12”, 12–16”, 16–20”, and 20–24” below surface). A fifth quid was analyzed from the 8–12” level to provide an additional replicate. Recognizing that the quid exteriors might have been contaminated from handling during the original excavation and subsequent museum storage, interior fibers from each specimen were subsampled into sterile 50 cc polypropylene centrifuge tubes for further analysis (**Fig 2**). Quid dissection and DNA extraction was conducted in a DRI laboratory not previously used for the processing of human tissue. Approximately 500 mg was removed from each quid for DNA extraction and an additional ~100 mg for radiocarbon dating (total quid mass averaged ~5 g). Quid sub-sampling was conducted with 95% ethanol-wiped-flamed utensils (sterilized tweezers/forceps/scissors) working on ethanol-soaked and flamed aluminum foil surface within a scrupulously cleaned, UV-treated Labconco Class II Biosafety Cabinet (Kansas City, MO) [43, 44]. While the DRI facility is not a certified clean room, precautions were taken during all laboratory manipulations to minimize potential contamination from handling (e.g. use of double nitrile gloves, lab coats).

DNA from each ~500 mg quid subsample was gently extracted in a sterile 15 mL polypropylene centrifuge tube using a modified phenol/chloroform method [7, 45, 46]. Briefly, the sample was submerged in 10 mL of lysis buffer (50 mM Tris (hydroxymethyl)-aminomethane (Tris), 50 mM disodium-ethylenediaminetetraacetic acid (EDTA), 150 mM NaCl, 2.5% w/v N-lauryl sarcosine, 500 mM 2-mercaptoethanol, 0.12 g proteinase K (30 U/mg, Invitrogen), vortexed for 10 seconds, and then incubated horizontally at 55^°^C for 24 hours with mild agitation at 80 rpm on a rotary shaker. After incubation, the lysate was removed from the insoluble quid remains using a sterile glass pipette and transferred to a new polypropylene centrifuge tube. The concentration of NaCl in the lysate was raised to 500 mM by adding an appropriate amount of 5M NaCl. In a fume hood pre-cleaned with 95% ethanol and flamed, the lysate was extracted three times with equal volume of phenol/chloroform/isoamyl alcohol (25:24:1). The extract was mixed for 15 minutes with gentle rocking, centrifuged at 4,000 x *g* for 10 minutes, and the organic phase was discarded. Lastly, the extract underwent a final extraction using an equal volume of chloroform followed by centrifugation at 4,000 x *g* for 10 minutes. The aqueous phase containing the DNA extract was purified using a QIAquick PCR Purification Kit (QIAGEN, Frederick, MD) as per company instructions. All precautions were taken to the best of our ability to meet proposed minimal ancient DNA analysis standards [47, 48].

### Human mitochondrial DNA amplification and sequencing analysis

Human mitochondrial DNA was amplified using 22 primer pairs which target 22 unique regions of the mtDNA genome (**S1 Table**) [7, 49]. Thermocycler conditions consisted of: an initial hot start (94°C for 5 min), 35 cycles of PCR (denaturation at 94°C for 30 seconds, annealing at 55°C for 30 seconds, and extension at 72°C for 1 minute), and a final extension step at 72°C for 4 minutes. GoTaq G2 polymerase (Promega, Madison, WI) was used according to manufacturer guidelines for PCR amplicon generation. To minimize primer bias, at least two independent amplifications were performed for each primer pair and the resulting amplicons were pooled. PCR products were screened by agarose gel electrophoresis (2%, TAE, 25 μM ethidium bromide) against a 1 kb ladder (Promega, Madison, WI) and products of the expected sizes were sent to Functional Biosciences (Madison, WI) for Sanger sequencing. DNA sequences were assembled and quality checked using Sequencher 4.9 (Gene Codes, Ann Arbor, MI). The resulting sequences from PCR amplicons with the highest Sanger confidence scores were compared to the revised Cambridge Reference Sequence (mtDNA, rCRS) (NC_012920) [50] for identification of SNPs. Variants and automated indel calls were manually/visually confirmed directly from the sequences and corrected to avoid alignment errors. SNPs were identified and haplogroups were assigned using Haplogrep 2.0 (http://haplogrep.uibk.ac.at/) [51] with the assistance of Mitomap (http://www.mitomap.org/MITOMAP) [52] and PhyloTree (http://www.Phylotree.org) [53].

### Method blanks and controls

Positive and negative DNA controls and method blanks were processed using the same DNA extraction and human mtDNA amplification procedures as the quid subsamples, with the exception that the lysis incubation for the positive control was only 4 h. Control DNA samples were collected via cheek swabs. The positive DNA control came from researcher L.T.H., a Caucasian who was directly involved in all aspects of the quid sample processing, and the negative DNA control came from a living canine (*Canis lupus familiaris*). Method controls (reagent blanks) were also performed to verify that DNA extraction kits reagents did not contain amplifiable human DNA.

### Independent confirmation of SNP and haplogroup assignment

Seven quid sub-sample splits (average 200 mg) from the original twenty-one quids processed at DRI were sent to the University of Montana (UM) for independent confirmation of results [54, 55]. The UM Molecular Anthropology Laboratory is a controlled-access facility; wherein researchers are required to wear Tyvek clean suits, foot coverings, hair nets, face masks, arm coverings, and gloves to enter. All Facility work surfaces are bleached daily using a 50% household bleach solution, and between the processing of each sample. Additionally, germicidal UV lights are run each evening for 1–4 hours, which bathes all horizontal surfaces to aid in decontamination, and the room maintains a positively pressurized environment. Movement from a laboratory working with post-PCR product to the ancient DNA laboratory was not allowed at any time.

Quid sub-samples were transferred to UM in sterile 50 mL polypropylene conical tubes at ambient temperature. After receipt, the outside of each tube was wiped down with a 50% bleach solution, followed by the addition of 5 mL of EDTA (0.5 M, pH 8). Samples were incubated without shaking at room temperature for 48 hours, after which 20 μL of 1 mg/mL Proteinase K (Invitrogen) was added to each, followed by sealing with parafilm and further incubation vertically at 52°C with slow rotation (6 turns/minute) for four hours. Once the samples were removed from incubation, they were extracted following the Dabney *et al*. protocol [56]. This entailed spinning the sample to the bottom of the tube by centrifugation at 1,500 x *g*, after which, the top 1.5 mL of the EDTA extract was pipetted into a new sterile, UV-treated 15 mL polypropylene tube. Next, 13 mL of PB Buffer (Qiagen) was added to each sample and mixed by inversion. The liquid was centrifuged at ~500 x *g* for 5 minutes through Qiagen MinElute filters utilizing 50 mL polypropylene tubes and nested conical reservoirs with attached filters. These filters were then removed, placed into a new collection tube, washed twice with PE Buffer (Qiagen), and eluted by centrifugation at 2000 x *g* with two 50 μL DNAse free H_2_O rinses into sterile, low-bind 2 mL tubes. An EDTA blank negative control was run through all the previous and following steps, and in no instance was contamination in subsequent PCR amplifications noted.

Following extraction, the samples were amplified using primers designed to target the Restriction Fragment Length Polymorphism (RFLP) markers diagnostic to each haplogroup, as well as the 9 base-pair deletion diagnostic of haplogroup B [57]. RFLP analysis was carried out in the UM Modern DNA room (a separate laboratory in a physically isolated, distant part of the building where PCR and analysis is carried out) following amplification and electrophoretic confirmation on a 1.5% w/v agarose gel utilizing ethidium bromide staining.

Amplification of the hypervariable region of the mtDNA was also completed utilizing four overlapping primer sets [58]. Sanger Sequencing was completed by McLab (San Francisco, CA). Sequence data were analyzed utilizing Sequencher (Gene Codes), and compared to the revised Cambridge Reference Sequence (mtDNA, rCRS) (NC_012920) [50] for identification of SNPs. SNPs were identified and haplogroups were assigned using Haplogrep 2.0 (http://haplogrep.uibk.ac.at/) [51], with the assistance of Mitomap (http://www.mitomap.org/MITOMAP) [52] and PhyloTree (http://www.Phylotree.org) [53].

### Radiocarbon analysis

Subsamples of approximately 100 mg from each quid were sent to DirectAMS (Bothell, WA) for analysis of radiocarbon content via accelerator mass spectrometry. Subsamples of four quids (#s 1, 11, 15, and 19) were also submitted to Beta Analytic (Miami, FL) for independent analysis of quid radiocarbon ages. At each facility (DirectAMS and Beta Analytic), quid fibers were subjected to an acid-base-acid pretreatment to remove inorganic carbon, soil humic acids, and superficially bound carbon. Conventional radiocarbon ages YBP were calculated by those facilities using fraction modern results corrected for isotopic fractionation using the concurrently measured δ^13^C of each graphitized sample. YBP radiocarbon dates were then calibrated via OxCal 4.3 (build number 104), employing the IntCal13 calibration curve for each sample, to obtain the reported ‘calibrated years before present’ (cal YBP) dates.

## Results

Twenty-one randomly selected quids (**Fig 2B**) were extracted for total DNA with each producing a minimum of 100 ng. DNA from all 21 quids was successfully amplified by PCR using 22 human mitochondrial (hu-mtDNA) primer pairs for possible 54% coverage of the mitochondrial genome [49]. With the exception of the modern hu-mtDNA control (**S2 Table**), none of the DNA extracts produced PCR products from all 22 primer pairs. Rather, coverage varied, ranging from 11.4% to 40.7% ($\bar{x}$ 25%). Successful PCR amplification of mitochondrial DNA did not reveal any correlation or bias to quid condition, depth of recovery, or radiocarbon age. The Sanger sequencing of the PCR products from each quid produced nucleotide identification for quids #3, 4, 5, 7, 8, 9, 10, 12, 13, 14, 17, 18, 19, and 21 which correlated with haplogroups C1c, D1, C1c, C1c, C1c, B2i1, C1c, C1c, C1c, C1c2, C1c, C1c, B2a5, and C1c, respectively (**Table 1**). Quids #1, 2, 6, 15, 16, and 20, Sanger sequence analysis revealed double chromatogram peaks at diagnostic Native American SNP positions. As a result, these quids haplogroup designations by DRI analysis were removed from the dataset, while their carbon dating results were kept as part of the dataset (**Table 1)**.

**Table 1:** Mule Spring Rockshelter quid haplogroups.

Quid No.	Strata Depth	Age		Diagnostic Native American mtDNA haplogroup(s) detected										Native American Supporting SNPs for Haplogroup Categorization
		Cal. YBP^1^ Range	% Interval^2^	A2		B2		C1		D1		X2a		
				DRI	UM†	DRI	UM†	DRI	UM†	DRI	UM†	DRI	UM†	
Control	n/a	n/a	**-**	**-**	**-**	**-**	**-**	**-**	**-**	**-**	**-**	**-**	**-**	-
1	0–4"	347–460	64.6	**-**	**-**	*	**B2**	**-**	**-**	**-**	**-**	**-**	**-**	DRI: A3547G, T4977C, C11177T, A11884G, C16278T
		348–458^	64.6											UM: C16111T, T16217C, C16278T, T16189C
2	0–4"	729–803.	71.5	**-**	**-**	**-**	**-**	*	-	**-**	**-**	**-**	**-**	DRI: G1888A,T16325C, C16327T
3	0–4"	693–796	94.8	**-**	**-**	**-**	**-**	**C1c**	**C1**	**-**	**-**	**-**	**-**	DRI: 249del, G1888A, T3552A, A9545G, G11914A, T16325C, C16327T
														UM: T16298C, T16325C, C16327T
4	0–4"	519–560	63.4	**-**	**-**	**-**	**-**	**-**	**-**	**D1**	**D**	**-**	**-**	DRI: G3010A, C5178A, C14668T, T16325C, T16362C
														UM: C16223T, G16319A, T16325C
5	8–12"	688–780	95.4	**-**	**-**	**-**	**-**	**C1c**	-	**-**	**-**	**-**	**-**	DRI: G3010A, G11914A, T14318C
6	8–12"	681–767	95.4	**-**	**-**	**-**	**-**	*	-	**-**	**-**	**-**	**-**	DRI: G1888A, G3010A, G11914A, T14318C, T16325C, C16327T
7	8–12"	675–760	95.4	**-**	**-**	**-**	**-**	**C1c**	-	**-**	**-**	**-**	**-**	DRI: 248del, 290-291del, G1888A, A9545G, G11914A, T14318C, T16325C, C16327T
8	8–12"	795–885	65.4	**-**	**-**	**-**	**-**	**C1c**	-	**-**	**-**	**-**	**-**	DRI: 248del, 290-291del, G1888A, T3552A, A9545G, T16325C, C16327T
9	8–12"	795–923	95.4	**-**	**-**	**B2i1**	-	**-**	-	**-**	**-**	**-**	**-**	DRI: C11177T, T16217C, T16311C
10	12–16"	737–833	56.1	**-**	**-**	**-**	**-**	**C1c**	-	**-**	**-**	**-**	**-**	DRI: 248del, 290-291del, G1888A, G11914A, T16325C, C16327T
11	12–16"	732–833	62.9	**-**	**-**	**-**	**-**	**C**^**‡**^	-	**-**	**-**	**-**	**-**	DRI: A9545G
		685–785^	95.4											
12	12–16"	669–729	95.4	**-**	**-**	**-**	**-**	**C1c**	**C1**	**-**	**-**	**-**	**-**	DRI: A249del, 290-291del, G1888A, T3552A, G11914A, T16325C, C16327T
														UM: C16223T, T16298C, T16325C, C16327T
13	12–16"	741–834	50	**-**	**-**	**-**	**-**	**C1c**	-	**-**	**-**	**-**	**-**	DRI: A249del, 290-291del, G1888A, A9545G, G11914A, T16325C, C16327T
14	16–20"	785–917	95.4	**-**	**-**	**-**	**-**	**C1c2**	-	**-**	**-**	**-**	**-**	DRI: G3010A, T16325C, C16327T
15	16–20"	910–977	94.8	**-**	**-**	**-**	**-**	*	**C1**	**-**	**-**	**-**	**-**	DRI: G1888A, G3010A, G11914A, T16325C, C16327T
		685–785^	95.4											UM: C16223T, T16298C, T16325C, C16327T
16	16–20"	725–799	88.6	**-**	**-**	**-**	**-**	*	-	**-**	**-**	**-**	**-**	DRI: T16325C, C16327T
17	16–20"	724–799	87.7	**-**	**-**	**-**	**-**	**C1c**	/	** **	**-**	**-**	**-**	DRI: 248del, 290-291del, G1888A, A9545G, G11914A, T16325C, C16327T
18	20–24"	795–883	58.8	**-**	**-**	**-**	**-**	**C1c**	-	**-**	**-**	**-**	**-**	DRI: G1888A, T16325C, C16327T
19	20–24"	795–921	95.4	**-**	**-**	**B2a5**	**-**	**-**	/	**-**	**-**	**-**	**-**	DRI: A3547G, G4820A, T4977C, C11172T, A11884G, T16217C, C16278T
		700–830^	78											
20	20–24"	728–831	73.5	**-**	**-**	**-**	**-**	*	-	**-**	**-**	**-**	**-**	DRI: G1888A, G3010A, T3552A, G11914A, T16325C, C16327T
21	20–24"	785–920	95.4	**-**	**-**	**-**	**-**	**C1c**	-	**-**	**-**	**-**	**-**	DRI: G1888A, G11914A, T16325C, C16327T

1 = Calibrated range in years before present.

2 = Percent confidence for most probable interval (%).

* = Competing sequences with non- and Native American haplogroups.

‡ = Only one SNP could be identified resulting in haplogroup C.

^ = Radiocarbon dates processed by BetaAnalytic, all others processed by DirectAMS.

† = University of Montana (UM), second confirmation analysis results.

DRI = Desert Research Institute.

UM = University of Montana.

/ = No haplogroup could be determined.

For quid #11, the only discernable Native American SNP was A9545G, therefore assigning a haplogroup with more detail than C was not possible. Quality assessment by Haplogrep 2.0 for haplogroup assignments averaged 0.586, with a low of 0.458 (Quid 9) and a high of 0.725 (Quid 19) (**S2 Table**). Haplogroups A2 and X2a were not observed in this sample set.

A subset of seven quid samples (#1, 3, 4, 12, 15, 17, and 19) from the original twenty one were sent to the Molecular Anthropology Laboratory at University of Montana for independent verification of the haplogroup identifications. From the seven samples, it was possible to confirm the haplogroup for five of the quids (71%) through RFLP and sequencing analysis. DNA was amplified from the remaining two samples (#17 and #19); however, the lack of diagnostic SNPs in the sequences prevented haplogroup assignment (there was no indication of contamination as a cause for not acquiring SNPs). For quids #1 and 15, independent analysis produced haplogroup assignments where Sanger sequencing was inconclusive due to double chromatogram peaks.

Radiocarbon analysis of a single fiber from each quid by DirectAMS yielded a range of radiocarbon ages from 347 to 977 cal YBP (**Table 1**). A subset of four samples, analyzed in parallel by Beta Analytic, ranged from 348 to 830 cal YBP. The age range for a given sample between the two labs was similar (102 years for Direct AMS vs. 110 years for Beta Analytic); but in all cases the median Direct AMS age was either in agreement (Quid 1) or older than that obtained by Beta Analytic (by an average of 88 years across four confirmatory quids). The most recent age (347–460 cal. YBP) correlated with quid #1, which was recovered from the shallowest stratification 0–4”. The ages generally trended towards progressively greater age with deeper recovery depths, although with sample to sample variation and not in a linear fashion.

## Discussion

Minimal genetic evaluation has been performed on museum cultural artifact assemblages from North America’s southern Great Basin. This study was motivated by the possibility that quids recovered from MSR and stored for many years in a museum collection might serve as a time-resolved genetic record for a regionally prominent archaeological site, as demonstrated by LeBlanc *et al*. for other sites [3]. In spite of a wide range of potential mechanisms for post-deposition contamination that might be expected for quids stored for fifty years, Native American haplogroups were successfully detected in all of the 21 quids examined in this pilot study. Hence, neither unknowable prehistoric events (e.g. bioturbation, DNA degradation, contamination from other quids or animal and human wastes), nor relatively modern impacts (e.g. looting and archaeological excavation conducted without concern for DNA), nor 50 years of room temperature storage negated the genetic utility of any of these samples. The careful incorporation and preparation of BSL-2 clean technique and tractable controls, consistent with suggested ancient DNA molecular biology best practices [47, 48] was sufficient to obtain useful genetic information from a very common type of archaeological sample.

Ultimately, this work revealed SNPs from mtDNA PCR amplicons that could be assigned to six known Native American haplogroup subclades (**Table 1**). Quality assessment scores by Haplogrep 2.0 are low because the primer sets selected for this study did not provide complete coverage of the mitochondrial genome (54% possible, **S2 Table**). In this dataset, coverage averaged 26%; thus, PCR amplification was variable and never 100% efficient. Correspondingly, we infer that that differences in amplification success between samples likely relates to random or regional vagaries in the preservation of mitochondrial DNA. However, as the acceptable PCR amplicon length of ancient DNA for analysis is typically ~150 nucleotides, though 500 nucleotides is also possible and acceptable, the somewhat greater lengths achieved with DNA extracted from quids at DRI (average ~350 nucleotides), suggests that the DNA was in relatively good condition [48]. Since no DNA stability study of human mitochondrial DNA preservation within quids has been completed, it is currently unclear if human cells desiccated in association with plant fibers would improve the preservation of genetic information over time, thus increasing the chances of obtaining longer PCR amplicons. Additional support for the conclusions of the main study comes from the samples sent to the Molecular Anthropology Laboratory at University of Montana for independent confirmation, where haplogroup designations of 71% of a subset of samples were confirmed.

To better constrain potential contaminating influence from individuals who may have contacted these samples, either prior to or during excavation in the 1960s/1970s or during the 50-year storage phase, mtDNA from the primary worker who conducted the laboratory work (of Bulgarian/Caucasian descent) was sequenced in parallel but not in the same pipeline with the samples from this collection as a control. With the exception of SNP G3010A, no Native American associated SNPs were detected in the control (**S3 Table**). SNP G3010A is known to be associated with haplogroups D4 and C1c2, which could potentially derive from contaminating DNA in this dataset. Only Quid 1 exhibited a SNP variation between two chromatograms of the SNP position that would have placed it either A2 or B2a5. This may have occurred through close contact with another quid. It is uncertain whether any further sample or equipment preparation could have improved the quality of DNA sequences. Although care was taken to extract internal core fibers, contamination cannot be ruled out and is potentially the cause of “compounded haplogroups” (double peaks) at diagnostic Native American SNP positions in our analysis. Quid 1 was sent for independent verification, revealing that it carried the 9 base-pair deletion indicative of haplogroup B, and HVRI sequence variation that also supports a haplogroup B designation.

The haplogroup results from this study can be assigned to the major pan-American lineages, with the majority being categorized as haplogroup C1c [27, 35, 59]. Prior molecular research shows that haplogroups C and D are the two most frequently encountered throughout central, northern, and eastern Asia, and the Americas [60]. The subclades of C1 (C1c and C1c2) are distinctly Native American, arising either during or sometime after humans crossed Beringia [27, 31, 60]. Although Haplogroup C1 is found throughout North, Central, and South America, the dominance of haplogroup C1 does not seem to be indicated by other ancient DNA studies of the Great Basin within the temporal span of our dataset [3, 27, 31, 34]. Rather, it appears that a maternal lineage that possessed subclades C1c and C1c2 routinely visited MSR or that an individual or group belonging to this type deposited a higher number of quids than other visitors during each time point, thus increasing the proportion of quids of the C1 haplogroup. The subclade B2a5 frequency distribution is centered on the Southwest and is hypothesized to have spread with the increased usage of agriculture into the Great Basin in the late Holocene [35]. In this dataset the Southwest haplogroup B2a5 appears only in the youngest and one of the older quids in the sample set (#1 and #19, 347–460 and 795–921 cal YBP, **Table 1**). One quid (#9, dated 795–923 cal YBP) contained human mtDNA belonging to haplogroup B2i1, which is curious because it is generally associated with South American populations [59]. Haplogroup D1 occurs only once in the data set, in the second youngest ^14^C-dated sample (#4, 519–560 cal YBP). The minimal presence of D1 and B2 lineages within our dataset results in an overall diminished quantity of diversity than expected from a random selection. The significance of these infrequently represented haplogroups within our time-resolved dataset is currently not understood, although this suggests that broader representation could be obtained within the total sum of recovered quids from MSR.

As was noted in the 1978 Turner thesis and surviving field notes, bioturbation was observed during the excavations and vandals further violated the site’s stratigraphy [1]. It is also possible that prehistoric activities in the cave resulted in ancient redistribution of shelter floor deposits. Thus, the use of arbitrary 3-D positional units for excavation in the 1960s/70s does not necessarily provide even relative chrono-stratigraphic context at MSR. In this study, direct ^14^C dating of sample splits was employed to assess stratigraphic integrity within the chosen study columns, but also to compensate for any of these known potential impacts to MSR’s process of deposition. While there is some discrepancy between the DirectAMS and BetaAnalytics results (**Table 1**), almost all of the quids (19/21) were dated ~600 to 980 cal YBP. Two of the relatively old quids (i.e. #2, 3; at 729–803 and 693–796 cal YBP, respectively) were found in the shallowest stratum (0–4”); while the somewhat younger quid (#12, at 669–729 cal YBP was recovered in the 12–16” depth). However, the seven quids with ages of greater than 750 years were relatively evenly distributed across the various levels; with multiple examples occurring in each of the 8–12”, 16–20”, and 20–24” strata. Further analysis of the remainder of the cave may in fact reveal that the deposits are more homogenous than anticipated, resulting from a more intensive use period with increased human-influenced depositional loads around ~600 to 980 cal YBP.

The age range of the full MSR artifact assemblage is also broader than that of the quid samples examined here. While the oldest quid of this dataset (collected at 16–20” depth, quid #15) correlates with an age of 910–977 cal YBP (or possibly 685–785 cal YBP, **Table 1**), four charcoal samples processed in the 1970s (it is unknown if these dates are calibrated) yielded results of 380 YBP (4”-8” depth), 1,125 YBP (12”-16” depth), 1,215 YBP (20”-24” depth), and 1,685 YBP (20”-24” depth) [1]. The upper three levels (0 to 12” below surface) across the shelter contained the bulk of the artifacts, including the ceramics. Temporally diagnostic pottery types range from Basketmaker III (circa 1,400 YBP), to Pueblo II (ending circa 850 YBP) and Great Basin Brown Ware (circa 1,000 YBP to historic times). Turquoise, shell, bone, and glass beads also primarily occur in the upper three levels and represent post-Archaic Period use of the shelter, with the glass beads reflecting post-European contact. Cottonwood and Desert Side-Notched types were the most abundant (60%) of the projectile points (circa 850 YBP to historic times) with lesser numbers (31%) of Rosegate (circa 1,450 YBP to circa 750 YBP). Archaic varieties (Elko, Humboldt, and Pinto) were recovered in 4” to 16” depth and comprise the remaining 9% of diagnostic point styles [1]. The bulk of the >4,000 quids (84%) were recovered from the surface to 16”-20” depth, similar to the other artifacts. As the current study only examined quids from 2 of the 150 excavated units which supplied the complete artifact collection, it is possible that older quids do exist in the collection and would be recognized upon more comprehensive examination.

While a depth-resolved timeline of habitation is limited by aforementioned disturbance at the original site and overlap of dating results, general distribution of ^14^C dates seem to indicate a concentrated presence of haplogroup C1c populations over the main time interval of this sample set (669 to ~977 cal YBP). Consistent with this observation is the fact that the two quids with disproportionately recent ages (#1 at 347–460 cal YBP and #4 at 519–560 cal YBP) appear to lack any affiliation with the C1c haplogroup.

It is tantalizing to suggest the large concentration of C1/C1c haplogroup quids in this dataset, from ~669 to 977 cal YBP at the Southwestern edge of the Great Basin, may be consistent with the linguistically supported Numic Spread hypothesis. This hypothesis proposes a rapid cultural expansion from the Owens Valley in California into the Great Basin approximately 700–950 YBP [61, 62]. The large number of quids preserved at MSR is unique among cave sites in the region. The dominance of haplogroup C1 over a ~300 year timeframe as suggested by the data is notable (**Table 1**). From this, it is possible to suggest that haplogroup C1 entered the region with Numic speakers, and a family of these individuals deposited a large volume of quids in MSR. Haplogroup C has been established as rare in the region prior to the Numic Spread [47]. However, extrapolation of a small number of DNA results from a single location to a larger regional context must be conducted with a great deal of caution.

As previously discussed, only 21 quids (~0.5% of the total) were selected from two adjoining units. Patterns of quid deposition could, for example, reflect bias if the quids were expelled in discrete pockets rather than uniformly scattered by shelter occupants. A more diverse sample of quids distributed both horizontally and vertically through the remainder of the cave deposits could further corroborate the concentration of haplogroup C1 during an intensive 300 year period of MSR use, or it could show a broader chronology and the presence of other haplotypes.

In the absence of a larger sample set (additional MSR quids or other regional caves), the genetic data obtained in this study cannot differentiate between the presence of a long-term resident population, migratory groups passing through, individuals who intentionally traveled to the cave as a periodic destination, or a massive cultural shift.

The combination of radiocarbon dates and mtDNA extracted from archived quid samples provides a convincing method for studying prehistoric habitation/visitation patterns at MSR. Examination of a larger sample set, with a more representative spatial distribution from the 1960s/70s excavation units would augment the results presented here. It could further address the paucity of time-resolved haplotype analyses from archaeological samples connecting MSR to California and the Great Basin. This study demonstrates the effectiveness of employing seldom-utilized but commonly archived materials to provide a wealth of information that reveals genetic details of southwest habitation locations that could provide important insights into ancient population interactions.

## Supporting information

S1 Table Human mitochondrial primer pairs. (XLSX)

S2 Table Summary of quid characteristics. (XLSX)

S3 Table Observed single nucleotide polymorphisms from quid PCR amplifications. (XLSX)
